# Sarcopenia, a Risk Predictor of Postoperative Acute Kidney Injury in Elderly Patients after Hip Fracture Surgery: A Retrospective Analysis

**DOI:** 10.3390/medicina60050745

**Published:** 2024-04-29

**Authors:** Seong Yoon Koh, Joo Hyun Jun, Jung Eun Kim, Mi Hwa Chung, Jihyo Hwang, Hye Sun Lee, Youngbum Jo, Eun Hee Chun

**Affiliations:** 1 Department of Anesthesiology and Pain Medicine, Kangnam Sacred Heart Hospital, Hallym University College of Medicine, Shingil-ro, Yeongdeungpo-gu, Seoul 07441, Republic of Korea; 2 Department of Orthopedics, Kangnam Sacred Heart Hospital, Hallym University College of Medicine, Seoul 07441, Republic of Korea; 3 Department of Biostatistics, Yonsei University College of Medicine, Seoul 06229, Republic of Korea

**Keywords:** acute kidney injuries, hip fractures, sarcopenia

## Abstract

*Background and Objectives:* Hip fracture surgery, which affects quality of life, can be a major challenge in geriatric populations. Although sarcopenia is known to be associated with postoperative outcomes, there are few studies on the association between sarcopenia and postoperative acute kidney injury (AKI) in this population. We investigated the association between sarcopenia and postoperative AKI in elderly patients following hip fracture surgery. *Materials and Methods:* We retrospectively reviewed the records of patients who underwent hip fracture surgery at our institution from March 2019 to December 2021. Patients under the age of 65, patients with no preoperative computed tomography (CT) scans and patients with inappropriate cross-sectional images for measurement were excluded. The psoas-lumbar vertebral index (PLVI), which is the ratio of the average area of both psoas muscles to the area of the fourth lumbar vertebral body, was measured from preoperative CT scans. Sarcopenia was defined as a PLVI within the lowest 25% for each sex, and patients were categorized into sarcopenic and nonsarcopenic groups. The occurrence of AKI was determined based on the serum creatinine level within postoperative day 7 using the Kidney Disease Improving Global Outcomes (KDIGO) guidelines. Univariate and multivariate logistic regression analyses were performed to evaluate the associations between clinical variables and the occurrence of AKI. *Results:* Among the 348 enrolled patients, 92 patients were excluded, and 256 patients were analyzed. The PLVI cutoff values for defining sarcopenia lower than 25% for male and female patients were 0.57 and 0.43, respectively. The overall incidence of AKI was 18.4% (47 patients), and AKI occurred more frequently in sarcopenic patients than in nonsarcopenic patients (29.7% vs. 14.6%, *p* = 0.007). According to the multivariate logistic regression, which included all variables with a *p* value < 0.05 in the univariate analysis and adjusted for age, body mass index (BMI) and American Society of Anesthesiologists (ASA) physical status, sarcopenia was revealed to be an independent predictor of postoperative AKI (odds ratio = 5.10, 95% confidence interval = 1.77–14.77; *p* = 0.003). *Conclusions:* Preoperative sarcopenia, which corresponds to the lowest quartile of PLVI values, is associated with postoperative AKI among elderly patients who underwent hip fracture surgery.

## 1. Introduction

The majority of hip fracture patients are elderly, and the complexity of their comorbidities and the degree of frailty vary. Performing surgery and anesthesia for these geriatric patients is challenging for both surgeons and anesthesiologists. There are significant concerns about elderly hip fracture patients because of the increased rate of mortality, inability to return to prior living circumstances and decreased quality of life [1]. Even a brief event can lead to catastrophic consequences for these vulnerable people.

Postoperative complications can cause delayed recovery and prolonged hospitalization. In particular, postoperative acute kidney injury (AKI) is the most common form of organ dysfunction; it affects up to 20% of patients during the early postoperative period and is known to have a great impact on multiorgan failure and mortality after hip surgery [2,3,4].

Sarcopenia, which may be interpreted as an age-related loss of muscle mass, is an objective and comprehensive marker of frailty [5,6]. Sarcopenia is studied mainly in cancer patients and is associated with mortality after oncological surgery and several other surgical conditions [7,8,9,10], including hip fracture surgery [11]. However, there is limited research on sarcopenia and the occurrence of AKI in elderly hip fracture patients.

To diagnose conventional sarcopenia, patients need to be able to move and cooperate well. However, it is impossible to perform these examinations in hip fracture conditions. Therefore, an alternative, easily obtainable method is needed. Central sarcopenia, as measured by the psoas cross-sectional area radiographically, was reported to be associated with poor prognosis in terms of morbidity and mortality in elderly trauma patients [11,12,13]. Therefore, we evaluated central sarcopenia using preoperative computed tomography (CT).

We investigated the association between sarcopenia and postoperative AKI in elderly patients following hip fracture surgery and determined other factors that could affect the occurrence of AKI.

## 2. Methods

**Figure 1:**
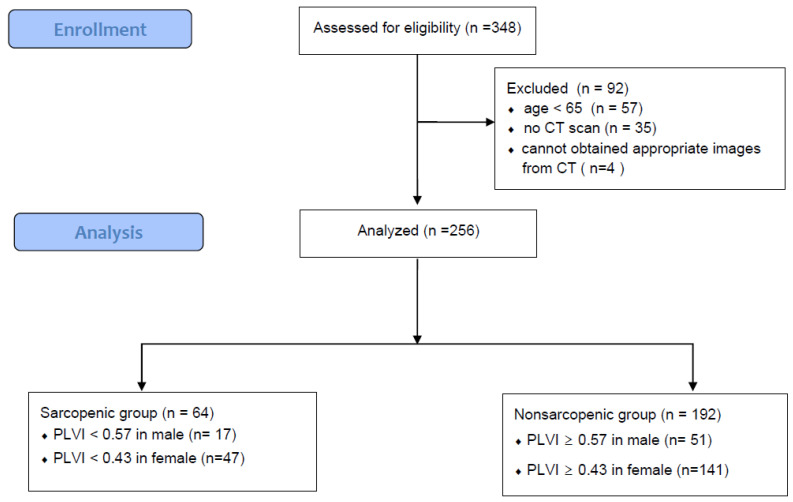
Flow chart.

The present study was conducted retrospectively at Kangnam Sacred Heart Hospital. Patients who underwent surgery for hip fracture from March 2019 to February 2021 were included. This study was approved, and informed consent was waived by the Kangnam Sacred Heart Hospital Institutional Review Board (HKS 2023-01-020) due to being a retrospective study. The exclusion criteria were (1) age younger than 65 years, (2) lack of preoperative CT scans and (3) inappropriate cross-sectional images for measurement (Figure 1).

Preoperative clinical data, operation-associated variables and postoperative outcomes were collected from the patients’ electronic medical records. Preoperative clinical data included age, sex, height, weight, body mass index (BMI), ideal body weight (IBW), American Society of Anesthesiologists (ASA) physical status and mechanism of injury and underlying conditions such as diabetes, hypertension, coronary artery occlusive disease (CAOD), chronic obstructive pulmonary disease (COPD) and chronic kidney disease (CKD). The medical conditions were confirmed through internal medicine consultation for preoperative evaluation if the patient had any history or abnormal laboratory findings. In case of CKD, if estimated glomerular filtration rate (eGFR) was less than 60 mL/min/1.73 m^2^, consultation was taken to nephrologist. We recorded any history of stroke or dementia, smoking or alcohol consumption or the use of beta-blockers or statins. Preoperative laboratory test data, including hemoglobin, albumin, creatinine and eGFR, were obtained.

The operation-associated variables included anesthesia methods (general or regional), operation and anesthesia time, crystalloid volume, colloid, packed red blood cells and fresh frozen plasma administered during surgery, diuretic use, urine output, overall intake and output and estimated blood loss. Postoperative outcomes, including AKI incidence, length of intensive care unit (ICU) stay, length of hospital stay, mortality within a month and blood transfusion, were also recorded.

The primary outcomes of the present study were the occurrence of postoperative AKI and the risk factors associated with AKI in elderly patients who underwent hip fracture surgery. The secondary outcomes were other perioperative variables, which were compared between the sarcopenia group and the nonsarcopenia group.

### 2.1. Sarcopenia Criteria

**Figure 2:**
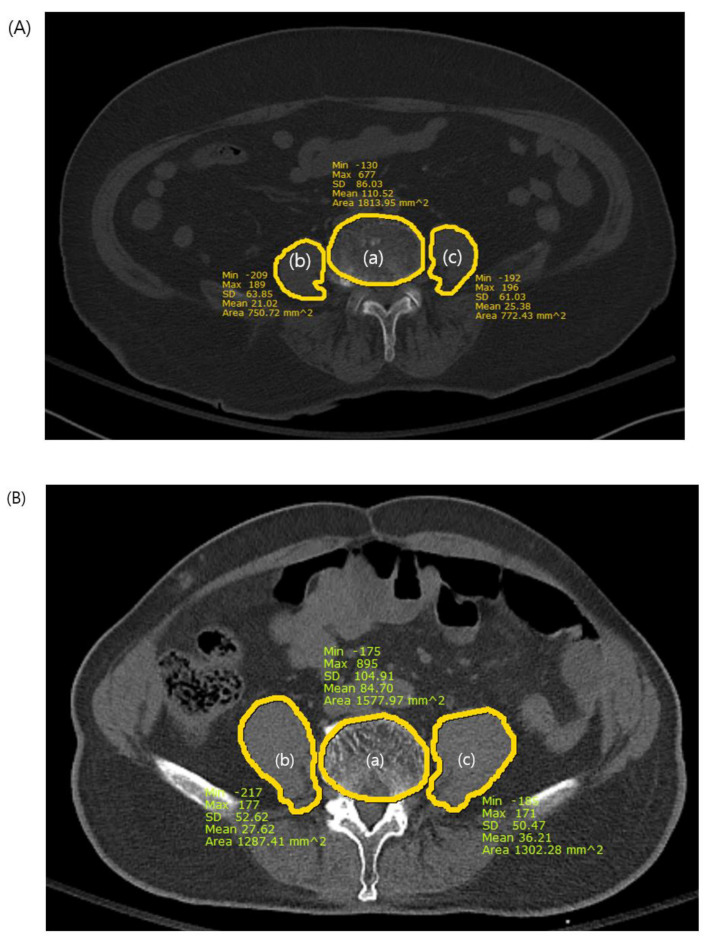
The psoas-lumbar vertebral index (PLVI) measurement. (a) L4 vertebral body area, (b) right psoas area, (c) left psoas area. PLVI = [(b + c)/2]/a (**A**): PLVI of the sarcopenic patient on computed tomography. The PLVI= 0.41. (**B**): PLVI of the nonsarcopenic patient on computed tomography. The PLVI = 0.84.

We measured the psoas-lumbar vertebral index (PLVI) to evaluate central sarcopenia from axial sections of preoperative CT images using measurements from the picture archiving and communication system (PACS). The PLVI was established following the criteria established in previous studies [11,12,13] and was calculated as the ratio of the average of the areas of both the right and left psoas muscles at the level of the inferior endplate of the fourth lumbar vertebra to the area of the fourth lumbar vertebra (Figure 2). Sarcopenia was defined as a PLVI within the lowest 25% for each sex [7,9,10], and patients were categorized into sarcopenic and nonsarcopenic groups for analysis.

### 2.2. Postoperative AKI

According to the Kidney Disease Improving Global Outcomes (KDIGO) guidelines [14] (Table 1), AKI was defined as an increase in the serum creatinine concentration of 0.3 mg/dL or more within 48 h, or an increase of 1.5 times or more from baseline within a week. We obtained postoperative serum creatinine data within postoperative day 7. In many cases, urine output data were not recorded; therefore, only the serum creatinine concentration was used to define AKI. The AKI stage was also evaluated.

### 2.3. Statistical Analysis

Patients were divided into two groups according to the cutoff value for sarcopenia (within the lowest 25% for each sex) in the present study. The perioperative variables were compared between the groups. After the normality test was conducted, continuous variables are presented as the means and standard deviations (SDs) and were tested using an independent two-sample *t* test. Categorical variables are presented as numbers with percentages and were tested using the chi-square test or Fisher’s exact test. Univariate logistic regression analysis was performed for preoperative and intraoperative variables to identify potential risk factors for postoperative AKI. The multivariate logistic regression model included all variables with a *p* value < 0.05 in the univariate analysis and adjusted for age, BMI and ASA physical status. Firth correction was used for variables for which the number of observations, such as postoperative transfusion, mechanism of injury and anesthesia method, was critically low. A *p* value < 0.05 was considered to indicate statistical significance for all analyses. Statistical analysis was conducted using SAS version 9.4 (SAS Institute, Inc., Cary, NC, USA).

## 3. Results

Among the 348 enrolled patients, 92 patients were excluded and 256 patients were analyzed. The PLVI cutoff values for defining sarcopenia lower than 25% for male and female patients were 0.57 and 0.43, respectively. We classified the patients into a sarcopenia group (*n* = 64) and a nonsarcopenia group (*n* = 192) (Figure 1).

### 3.1. Demographic and Clinical Characteristics

The demographic data and preoperative laboratory test values of the patients are summarized in Table 2. The mean PLVI was 0.37 in the sarcopenia group and 0.63 in the nonsarcopenia group. The mean ages of the individuals in each group were 83.4 years and 79.2 years, respectively (*p* < 0.001). The patients were mainly female, and there was no difference in sex between the two groups. Weight was significantly different, with a mean of 53.9 kg in the sarcopenia group and 57.7 kg in the nonsarcopenia group. BMI was also significantly different (21.6 kg/m^2^ vs. 23.2 kg/m^2^, respectively). The distribution of patients with severe systemic disease, ASA physical status III, was the same in both groups.

The most common cause of fracture in both groups was slipping down on a flat surface, which corresponded to low-energy trauma. There were no significant differences between the two groups regarding the presence of diabetes mellitus, hypertension, CAOD, COPD, CKD, a history of stroke, dementia, smoking, alcohol consumption or the use of medication, beta-blockers or statins.

The mean hemoglobin levels determined via preoperative laboratory tests were 11.2 g/dL in sacrcopenic group and 11.7 g/dL in nonsarcopenic group. (*p* = 0.04); however, the clinical significance of these differences appears to be negligible. The mean serum albumin concentrations were 3.3 g/dL in sacrcopenic group and 3.7 g/dl in nonsarcopenic group. (*p* < 0.001). Hypoalbuminemia (<3.4 g/dL) was observed more frequently in the sarcopenia group (*p* = 0.048) than in the nonsarcopenia group.

### 3.2. Operation-Associated Variables 

Table 3 compares operation-associated variables between the groups. Most of the patients underwent general anesthesia; all patients in the sarcopenia group, and 92.2% of the patients in the nonsarcopenia group. Fifteen patients of the nonsarcopenia group underwent regional anesthesia. There was a significant difference in the methods of anesthesia between the two groups (*p* = 0.03). There were no significant differences in the duration of surgery or anesthesia between the two groups. With respect to intravascular volume optimization, including the volume of crystalloid and colloid administered, blood transfusion, administration of diuretics and total volume of intake and output, there were no significant differences during the operation between the two groups.

### 3.3. Postoperative Outcomes

Among the 256 patients, 47 developed postoperative AKI, and the overall incidence of postoperative AKI was 18.4%. In the sarcopenic group, 19 patients experienced postoperative AKI (29.7%), while in the nonsarcopenic group, 28 (14.6%) experienced postoperative AKI (Table 4). There was a significant difference between the two groups in terms of 1-month mortality (17.2% vs. 5.2%) (*p* = 0.003).

### 3.4. Univariate and Multivariate Models for AKI Incidence

The risk-adjusted outcomes according to the logistic regression model are presented in Table 5. Female sex, sarcopenia, ideal body weight, hypertension, CKD, hypoalbuminemia (<3.4 g/dL), preoperative serum creatinine, eGFR, operation time and intraoperative diuretic use were associated with the occurrence of postoperative AKI within 7 days according to univariate analysis (*p* < 0.05). 

According to the multivariate analysis, sarcopenia (odds ratio (OR) = 5.10, 95% confidence interval (CI) = 1.77–14.77, *p* = 0.003) based on the PLVI was identified as an independent predictor of postoperative AKI. Furthermore, the association between operation time and postoperative AKI also remained significant (OR = 1.02, 95% CI = 1.002–1.03, *p* = 0.03). The associations between AKI and several factors, including female sex, ideal body weight, hypertension, CKD, hypoalbuminemia, serum creatinine, eGFR and intraoperative diuretic use were not significant after adjustment for age, BMI, ASA status and the other significant (*p* < 0.05) variables in the univariate model. 

## 4. Discussion

### 4.1. Sarcopenia

The main findings of this study are as follows: (i) Sarcopenia defined by the preoperative PLVI value was a strong predictor of postoperative AKI in elderly patients who underwent hip fracture surgery; (ii) Other factors considered to be associated with AKI, especially hypoalbuminemia, were no longer significant factors according to the multivariate model after adjustment for age, BMI and ASA physical status and the other significant (*p* < 0.05) variables in the univariate model.

The diagnostic criteria for sarcopenia include three aspects: muscle mass, muscle strength and physical performance [6]. There has been growing research interest in the clinical application of sarcopenia in combination with frailty. Central sarcopenia based on the psoas muscle is associated with postoperative complications, mortality, hospital stay and costs in various surgical fields [7,8,9,10,11,12,13]. 

The methodology for evaluating central sarcopenia in morphometrics has not yet been established. Recent studies have suggested several methods for measuring the psoas muscle (for example, the sum of the total psoas area, the average of both psoas areas, the normalized psoas area by height or body surface area and the psoas-lumbar vertebral index [7,9,10,12,13,15]. Currently, there are no determined cutoff values for morphometric parameters, including the PLVI; therefore, the study-dependent cutoff values were proposed for central sarcopenia. Most of the studies set the cutoff values at the lowest quartile according to sex [9,10,13,15]. It is difficult to reach a consensus on the definition of the morphometric cutoff value for sarcopenia because the range of values varies depending on the patient group. Nevertheless, radiologically defined sarcopenia seems to be useful in terms of preoperative risk prediction and surgical decision-making.

### 4.2. Sarcopenia–AKI Mechanism 

The incidence of AKI in patients undergoing hip fracture surgery is reportedly 15%~24% [2], and in the present study, it was similarly observed to be 18.4%. The most common causes of death after hip fracture surgery are infection, such as pneumonia, cardiovascular events and AKI [16], and AKI is a significant factor affecting the morbidity and mortality of this population [2,3,4]. An observed association requires hypothesis testing to provide evidence, which includes evidence of a causal association [17], so we focused on several possible mechanisms of the association between sarcopenia and AKI.

First, the activation of inflammatory pathways due to chronic inflammation is speculated to be associated with sarcopenia [18,19]. Recent aging models have suggested an age-related, chronic state of low-grade inflammation, also known as “inflammaging” [20]. The skeletal muscle system is a complex influenced by numerous metabolic pathways. During a state of oxidative stress and chronic systemic inflammation, which remains typical of aging, skeletal muscle may exhibit exacerbated atrophy through mechanisms mediated particularly by tumor necrosis factor-alpha [19]. High risk of AKI could be attributed to inflammatory pathway activation leading to increased levels of inflammatory cytokines such as interleukin-6, interleukin-15 and tumor necrosis factor-alpha that tend to negatively affect renal function [6,19]. This may also explain the negative association between serum albumin, a marker of inflammation, and frailty and sarcopenia, regardless of participant age and setting [21].

Second, serum albumin was also suggested as a biomarker of frailty and sarcopenia [21] and a simple indicator of undernutrition [22]. Hypoalbuminemia has been well established as a potent independent risk factor for AKI [23]. Although the pathogenesis has not been fully proven, albumin plays a role in maintaining renal perfusion, glomerular filtration and endothelial functions [24]. The endothelial glycocalyx may be disrupted after severe trauma or surgery [25]. Likewise, endothelial damage following surgery leads to capillary leakage and hypoalbuminemia. It may worsen renal function and trigger AKI. Consequently, the serum albumin concentration is already in a state of change in acute trauma patients due to accompanying endothelial damage, so it may be difficult to establish baseline values in hip fracture patients. Therefore, predicting AKI based on sarcopenia using the PLVI has a greater advantage than relying on serum albumin, which is an inaccurate baseline measurement.

Third, the reduced amount of physical activity commonly observed in sarcopenia is accompanied by a decrease in cardiac output. Decreased cardiac output leads to a reduction in renal blood flow; accordingly, there is a decrease in glomerular filtration rate [26]. In sarcopenic patients, there is a decrease in renal function in the form of a reduction in the eGFR [27,28]. 

Fourth, sarcopenic patients tend to have increased adipose tissue and decreased total body water. The acute stress response to surgery includes secretion of antidiuretic hormone and increased water retention; an aging kidney with the changes within the tubule lead to a decreased ability to retain sodium. The elderly might be particularly vulnerable in times of stress involving larger volume shifts [26]. Sarcopenic patients have the previously mentioned characteristics of hypoalbuminemia, decreased eGFR, decreased cardiac output, decreased renal blood flow and decreased total body water, making them more vulnerable to volume control.

### 4.3. Other Explanations

There are conflicting reports regarding the outcomes of general anesthesia and regional anesthesia. A meta-analysis found that the AKI incidence was lower for regional anesthesia when compared with general anesthesia [29], but another study did not [30]. A sympathetic blockade of T4 -T10 levels via regional anesthesia may theoretically improve renal perfusion but it has been reported that epidural anesthesia does not change the renal blood flow for healthy volunteers [31]. Strong evidence supports similar outcomes for general or spinal anesthesia for patients undergoing hip fracture surgery in practice guidelines [1]. Since the general anesthetic drugs that may affect the kidneys have changed significantly nowadays and the equipment to ensure hemodynamic stability during regional anesthesia has been also improved, updated research will be needed.

Although the anesthesia method was significantly different between the two groups, the association between anesthesia method and postoperative AKI was not significant according to univariate analysis (OR = 0.13, 95% CI = 0.01–2.45, *p* = 0.17) (Table 5). Our institution does not have a different strategy on anesthesia methods depending on the degree of frailty. For elderly trauma patients, the anesthesia method was determined considering spine degenerative disease, difficulty of position change and patient preference. 

Previous studies have reported the association between AKI and several factors including anemia, preoperative serum creatinine, higher age, heart disease, comorbidities, obesity, hypoalbuminemia, blood transfusion and C-reactive protein; however, there was some discrepancy between reports [2,4,23,32,33,34,35]. The disagreement is assumed to result from the methodological differences including recognition of CKD, AKI definition and central sarcopenia criteria. The history of CKD was retrieved from medical records in the present study and there was no significant difference between the sarcopenia group and the nonsarcopenia group in preoperative serum creatinine and eGFR values (Table 2). However, concerns may exist regarding the use of creatinine-based equations as they may misestimate GFR in conditions in which creatinine formation is decreased, as in sarcopenic older adults. Because muscle mass is a major determinant in the generation of creatinine, measured GFR via inulin or iohexol clearance methods could be more appropriate in these patients [36]. Thus, it is possible that the subjects of this study also had overestimated GFR during their routine examinations and were not aware of CKD [37]. For these patients, complementary tests are recommended. The KDIGO group has previously updated the CKD guidelines by including proteinuria [38]. Therefore, in patients who may misestimate GFR, proteinuria can be used to detect CKD or as a predictor of surgery outcomes [39]. Additionally, there was insufficient information about urine output to determine AKI incidence, and muscle strength and physical performance among the sarcopenia criteria were also unknown.

Furthermore, operation time is associated with postoperative AKI. The operation time is related to the fracture type and operation methods, so further studies are needed. It is suggested that its impact on the occurrence of postoperative AKI is not substantial, considering that the odds ratio for operation time was 1.02. However, thoroughly considering methods to shorten the operation time whenever possible is worthwhile.

### 4.4. Practical Application 

Several measures for preventing AKI in patients undergoing hip fracture surgery are needed. It is necessary to identify AKI high-risk patients before surgery, and renal functions in all patients should be carefully evaluated. Special attention should be given to patients with underlying conditions such as sarcopenia, hypertension and CKD. While assessing the overall skeletal muscle mass, muscle strength and functional capacity of patients may be challenging, predicting whether a patient is in a sarcopenic state can be easily achieved through pelvic CT performed before surgery. The three most important principles are to maintain normovolemia, to avoid nephrotoxic drugs and to achieve hemodynamic stability [32]. The practical plans include sharing the information on risk of AKI among the staff and taking a team approach, minimizing blood loss, counting estimated blood loss accurately, meticulous perioperative fluid management, avoiding the use of nonsteroidal anti-inflammatory drugs and taking a multimodal approach of pain control. 

### 4.5. Limitations

This retrospective study has a limitation in using creatinine-based criteria for evaluation of kidney functions. It cannot establish a causal association [17] between sarcopenia and AKI, anesthesia method and AKI, so, it should be interpreted cautiously. Since there is previous research on relevant biological plausibility, further prospective studies including causal inference are required.

## 5. Conclusions

This study showed that preoperative sarcopenia, which corresponds to the lowest quartile of PLVI values, is associated with postoperative AKI. The major strength of this study is that it suggests the possibility of predicting AKI using the PLVI, without relying on an uncertain history of comorbidity or serum albumin, which makes it difficult to establish a baseline for trauma patients. Identifying patients, with attention given to this factor, may contribute to meticulous management for achieving better postoperative outcomes.

## Figures and Tables

**Table 1:** KDIGO staging for AKI [14].

Stage	Serum Creatinine
1	1.5–1.9 times baseline or ≥0.3 mg/dL (26.5 μmol/L) increase
2	2.0–2.9 times baseline
3	3 times baseline or ≥4.0 mg/dL (353.6 μmol/L) increase or initiation of RRT or in patients < 18 years a decrease in eGFR < 35 mL/min/1.73 m^2^

KDIGO = Kidney Disease Improving Global Outcomes; AKI = acute kidney injury; RRT = renal replacement therapy; eGFR = estimated glomerular filtration rate.

**Table 2:** Demographic and clinical characteristics.

Variable	Sarcopenic	Nonsarcopenic	*p*-Value
	(*n* = 64)	(*n* = 192)	
PLVI	0.37 ± 0.10	0.63 ± 0.13	<0.001 *
Total psoas area (mm^2^)	652.3 ± 221.8	889.2 ± 242.6	<0.001 *
Age (years)	83.4 ± 6.6	79.2 ± 7.6	<0.001 *
Sex (M/F)	17/47	51/141	>0.99
Height (cm)	157.6 ± 8.4	157.7 ± 7.7	0.94
Weight (kg)	53.9 ± 10.4	57.7 ± 10.6	0.01 *
BMI (kg/m^2^)	21.6 ± 3.7	23.2 ± 4.0	0.006 *
IBW (kg)	51.4 ± 9.2	51.6 ± 8.4	0.91
ASA physical status			0.07
I, II/III	60 (93.8)/4 (6.3)	189 (98.4)/3 (1.6)	
Mechanism of injury			0.67
Slip down	50 (78.1)	137 (71.4)	
Fall down within 1 m	9 (14.1)	39 (20.3)	
Fall down over 1 m	2 (3.1)	5 (2.6)	
Traffic accident	0 (0.0)	3 (1.6)	
Others	3 (4.7)	8 (4.2)	
Diabetes mellitus	19 (29.7)	75 (39.1)	0.18
Hypertension	50 (78.1)	131 (68.2)	0.13
CAOD	9 (14.1)	19 (9.9)	0.36
COPD	3 (4.7)	4 (2.1)	0.27
CKD	8 (12.5)	28 (14.6)	0.68
History of:			
stroke	13 (20.3)	45 (23.4)	0.61
dementia	18 (28.1)	33 (17.2)	0.06
smoking	1 (1.6)	3 (1.6)	>0.99
alcohol use	2 (3.1)	5 (2.6)	0.82
Patients taking:			
beta-blocker	0 (0.00)	1 (0.52)	0.56
statin	4 (6.25)	18 (9.38)	0.44
Preoperative value			
Hemoglobin (g/dL)	11.2 ± 2.0	11.7 ± 1.8	0.04 *
Albumin (g/dL)	3.3 ± 0.5	3.7 ± 0.5	<0.001 *
Albumin < 3.4 (g/dL)	25 (39.1)	50 (26.0)	0.048 *
Creatinine (mg/dL)	0.8 ± 0.4	0.9 ± 0.6	0.16
eGFR (ml/min/1.73 m^2^)	77.0 ± 19.7	73.5 ± 21.4	0.25
Calcium, total (mg/dL)			0.082
<8.4	18 (28.1)	30 (15.6)	.
Normal (8.4~10.2)	46 (71.9)	160(83.3)	.
>10.2	0 (0.0)	2 (1.0)	.
Phosphorus, total (mg/dL)			0.505
<2.6	8 (12.5)	32 (16.7)	.
Normal (2.6~4.6)	54 (84.4)	157 (81.8)	.
>4.6	2 (3.1)	3 (1.6)	.

The values are given as the mean ± standard deviations or as the number of patients, with the percentage in parentheses. PLVI = psoas-lumbar vertebral index; Total psoas area = the average of bilateral psoas area; BMI = body mass index; IBW = ideal body weight; ASA = American Society of Anesthesiologists; CAOD = coronary artery occlusive disease; COPD = chronic obstructive pulmonary disease; CKD = chronic kidney disease; eGFR = estimated glomerular filtration rate. *: *p* < 0.05.

**Table 3:** Operation-associated variables.

Variable	Sarcopenic	Nonsarcopenic	*p*-Value
	(*n* = 64)	(*n* = 192)	
Anesthesia			0.03 *
General/Regional	64 (100)/0 (0)	177 (92.2)/15 (7.8)	
Operation time (min)	58.7 ± 34.0	56.0 ± 24.9	0.63
Anesthesia time (min)	104.2 ± 31.5	108.0 ± 33.1	0.42
Crystalloid intake (mL)	669.3 ± 303.5	647.3 ± 283.9	0.60
Colloid intake (mL)	16.9 ± 75.2	29.4 ± 98.5	0.30
RBC transfusion (mL)	37.0 ± 89.1	31.8 ± 88.7	0.68
FFP transfusion (mL)	0	0	>0.99
Diuretics use (n)	2 (3.1)	9 (4.7)	0.74
Urine output (mL)	131.5 ± 197.3	148.6 ± 206.3	0.56
Total intake (mL)	723.0 ± 356.4	702.9 ± 321.0	0.67
Total output (mL)	251.3 ± 239.2	276.0 ± 253.2	0.50
Estimated blood loss (mL)	121.1 ± 113.8	128.0 ± 101.1	0.65

The values are given as the mean ± standard deviations or as the number of patients, with the percentage in parentheses. RBC = red blood cells; FFP = fresh frozen plasma. *: *p* < 0.05.

**Table 4:** Postoperative outcomes.

Variable	Sarcopenic	Nonsarcopenic	*p*-Value
	(*n* = 64)	(*n* = 192)	
AKI	19 (29.7)	28 (14.6)	0.007 *
Stage 1	13 (68.4)	20 (71.4)	0.83
Stage 2	4 (21.1)	6 (21.4)	>0.99
Stage 3	2 (10.5)	2 (7.1)	>0.99
Onset of AKI (postoperative days)	1.6 ± 0.6	1.2 ± 0.5	0.063
Duration of AKI (days)	11.3 ± 23.4	15.8 ± 24.5	0.564
ICU stay (days)	4.5 ± 11.6	2.0 ± 7.2	0.11
Hospital stay (days)	21.6 ± 14.8	21.7 ± 13.0	0.94
Mortality 1-month	11 (17.2)	10 (5.2)	0.003 *
Postoperative transfusion			0.27
Within a week	55 (85.9)	153 (79.7)	
Not done	9 (14.1)	39 (20.3)	
RRT	1(1.6)	0(0.0)	0.25

The values are given as the mean ± standard deviations or as the number of patients, with the percentage in parentheses. AKI = acute kidney injury; ICU = intensive care unit; RRT = renal replacement therapy. *: *p* < 0.05.

**Table 5:** Risk-adjusted outcomes for postoperative acute kidney injury.

Variable	Univariate Model		Multivariate Model	
	OR (95% CI)	*p*-Value	OR (95% CI)	*p*-Value
Age (years)	1.01 (0.97–1.05)	0.74	0.97 (0.91–1.04)	0.43
Sex (female)	1.97 (1.01–3.85)	0.046 *	2.96 (0.47–18.40)	0.25
PLVI	0.25 (0.04–1.73)	0.16		
Sarcopenia (PLVI < 25%ile)	2.47 (1.27–4.83)	0.008 *	5.10 (1.77–14.77)	0.003 *
Total psoas area (mm^2^)	1.00	0.43		
No postoperative transfusion	0.58 (0.23–1.46)	0.25		
Body mass index (kg/m^2^)	1.01(0.94–1.10)	0.73	0.99 (0.86–1.13)	0.85
Ideal body weight (kg)	1.04(1.01–1.08)	0.02 *	1.01 (0.92–1.11)	0.82
ASA physical status (III)	1.81 (0.34–9.65)	0.49	2.52 (0.29–22.0)	0.40
Mechanism of injury				
Fall down within 1 m	0.97 (0.43–2.15)	0.93		
Fall down over 1 m	0.27 (0.01–5.83)	0.40		
Traffic accident	2.41 (0.23–25.62)	0.47		
Others	0.17 (0.01–3.43)	0.25		
Diabetes mellitus	1.35 (0.71–2.57)	0.36		
Hypertension	2.30 (1.02–5.19)	0.045 *	1.38 (0.43–4.43)	0.59
CKD	4.88 (2.28–10.42)	<0.001 *	1.25 (0.27–5.78)	0.78
Preoperative laboratory test				
Hemoglobin (g/dL)	0.89 (0.75–1.05)	0.16		
Hypoalbuminemia (<3.4 g/dL)	2.32 (1.21–4.46)	0.01 *	1.58 (0.60–4.16)	0.35
Creatinine (mg/dL)	4.07 (2.17–7.65)	<0.001 *	0.82 (0.11–6.24)	0.85
Estimated GFR (mL/min/1.73 mm^2^)	0.97 (0.95–0.98)	<0.001 *	0.95 (0.90–1.00)	0.06
Anesthesia (regional)	0.13 (0.01–2.45)	0.17		
Intraoperative data				
Operation time (min)	1.01 (1.001–1.02)	0.04 *	1.02 (1.002–1.03)	0.03 *
Diuretic use	8.97 (2.51–32.08)	0.001 *	3.90 (0.58–26.34)	0.16
Urine output (mL)	1.02 (0.88– 1.19)	0.78		
Total intake (mL)	1.04 (0.95–1.14)	0.39		
Total output (mL)	1.00 (0.88–1.14)	0.98		
Estimated blood loss (mL)	0.96 (0.70–1.31)	0.77		
Calcium, total				
Normal (8.4–10.2)	0.60 (0.29–1.27)	0.18		
>10.2	0.58 (0.01–25.47)	0.78		
Phosphorus, total				
Normal (2.6–4.6)	0.73 (0.32–1.67)	0.46		
>4.6	0.86 (0.09–8.71)	0.90		

Multivariate logistic regression model included all variables with *p*-value of <0.05 in the univariate analysis and adjusted for age, BMI and ASA physical status. OR = odds ratio; CI = confidence interval. PLVI = psoas-lumbar vertebral index; ASA = American Society of Anesthesiologists; CKD = chronic kidney disease; eGFR = estimated glomerular filtration rate; EBL = estimated blood loss. *: *p* < 0.05.

## Data Availability

The datasets generated and/or analyzed during the current study are not publicly available due to the regulations of the Institutional Review Board, but are available from the corresponding author, after getting permission from IRB for sharing the datasets on reasonable request.

## List of Abbreviations

AKI: acute kidney injury; CT: computed tomography; PLVI: psoas-lumbar vertebral index; KDIGO: Kidney Disease Improving Global Outcomes; ASA: American Society of Anesthesiologists; BMI: body mass index; IBW: ideal body weight; CAOD: coronary artery occlusive disease; COPD: chronic obstructive pulmonary disease; CKD: chronic kidney disease; eGFR: estimated glomerular filtration rate; ICU: intensive care unit; RRT = renal replacement therapy; PACS: picture archiving and communication system; SD: standard deviations; OR: Odds ratio, CI: confidence interval.
